# Supplementary material for: Sustainability of translator training in higher education
Source: PLoS One. 2023 May 16;18(5):e0283522. doi: 10.1371/journal.pone.0283522 (PMC10187915; doi:10.1371/journal.pone.0283522)
Supplement: S1 Table — (PDF) [file pone.0283522.s001.pdf]

Item-Analysis

	Group (M±SD)		t(CR)	P
	LOW-Score Group (n=20)	High-Score Group (n=42)		
4、This course enables me to have some idea about transcreation.	1.40±0.50	2.05±0.31	5.306	0.0
5、Transcreation, a form of rewriting or copy-writing, deviates, to a lesser or greater extent, or even completely, from the source text, so as to better serve the target audience.	1.60±0.75	2.17±0.49	3.067	0.0
6、This course enables me to understand that human creativity can never be replaced by AI or machine translation when it comes to cross-cultural promotional especially advertising and marketing and other communicative purposes.	1.50±0.61	2.07±0.51	3.637	0.0
7、 Whether to adopt transcreation depends on the skopos or purpose of the translation involved.	1.25±0.44	2.24±0.58	6.760	0.0
8、 In translation, the end (skopos or purpose) justifies the means including but not limited to transcreation.	1.30±0.47	2.07±0.34	6.559	0.0
9、In the age of AI, transcreation is a core competence or skill for translators when most of conventional translation is taken over by AI or machine translation	1.05±0.22	2.14±0.42	10.953	0.0
10、 The popularity of AI or machine translation means a great opportunity for those with skills in transcreation or copy-writing.	1.25±0 55	2.12±0.55	5.815	0.0
11、This course has boosted my competitiveness or employability to some degree as a would-be translator.	1.40±0.60	2.17±0.44	5.118	0.0
12、The prospects of the job market in the translation industry are bright though facing huge challenges.	1.45±0.60	2.31±0.56	5.490	0.0
13、The effects of transcreations by students are hard to assess since the market has the final say even if translation teachers or clients are impressed.	1.35±0.49	2.07±0.26	6.188	0.0

*p<0.05 ** p<0.01

Suggestions

Item analysis is used to measure the validity and suitability of the items in the questionnaire. Its principle is that if the analysis items are designed reasonably, the samples aredifferentiated and some of the scores are high and some are low. If the design of scale items is unreasonable, it is likely that all groups choose the same answer, so such scale items need to be deleted or modified.

First, describe the basic principle and process of item analysis;

Secondly, the final result should be described and analyzed in depth, and whether the analysis items should be deleted or not;

Third: Summarize the analysis.

Intelligent Analysis

The purpose of project analysis is to determine whether the research items in the questionnaire is valid and appropriate. The principle is to sum the analysis items first and then divide them into high scores and low scores (bounded by 27% and 73% quantiles), and then use t-test.Check and compare the differences between the high score and the low score. If there are differences, it means that the scale items are designed properly.Otherwise, it means that the scale items cannot distinguish information and that the design is unreasonable, the items involved should be deleted.The analysis is based on the following 10 items:
4、 This course enables me to have some idea about transcreation.5、 Transcreation, a form of rewriting or copy-writing, deviates, to a lesser or greaterextent, or even completely, from the source text, so as to better serve the target audience.6, This course enables me to understand that human creativity cannever be replaced by AI or machine translation when it comes to cross-cultural promotional especially advertising and marketing and other communicative purposes.7、Whether to adopt transcreation depends on the skopos or purpose of the translation involved.8, In translation, the end (skopos or purpose) justifies the means including but not limited to transcreation. 9、In the age of AI, transcreation is a corecompetence orskill for translators when most of conventional translation is taken over by AI or machine translation.

10、The popularity of AI or machine translation meansagreat opportunity for those with skills in transcreation or copy-writing. 11, This course has boosted mycompetitiveness or employability to some degreeasawould-be translator.12、 The prospects of the job market in the translation industry are bright though facinghuge challenges.13, The effects of transcreationsbystudents are hard to assess since the market has the final say even if translation teachers or clients are impressed.
A total of 10 items are analyzed, and after summing these 10 items, they are divided into high-score and low-score groups, and the differences are compared by t-test. As can be seen from the above table, all of them are significantly different(p<0.05), which means that the 10 items are well differentiated and should be retained.It can be concluded that none of the 10 items below should be deleted.
