# Supplementary material for: Sustainability of translator training in higher education
Source: PLoS One. 2023 May 16;18(5):e0283522. doi: 10.1371/journal.pone.0283522 (PMC10187915; doi:10.1371/journal.pone.0283522)
Supplement: S1 Appendix — (PDF) [file pone.0283522.s002.pdf]

Company profile

Founded in 1971, headquartered in Zhongshan, Four Seas is a residential and hotel furniture provider. We manufacture high-quality standard home furniture, customized home furniture, interior doors, decoration panels, built-in walk-in wardrobe/ cabinets, and can offer a whole-house furnishings solution for our customers.

Four Seas has modern manufacturing base of more than 180,000 ㎡ , which is with international advanced production equipment. There are 70,000 ㎡ stereo modern warehousing distribution center to meet the demand of order of the company's global marketing network.

Over the past 50 years, Four Seas' export business has ranged to Asia, Europe, North America, South America and Africa etc., selling to more than 60 countries and regions. The distribution networks of overseas markets are including brand shop, distributor, exclusively agents etc, which could satisfy different consumers' demand and bring brilliant furnishings cultural experience to the global customers.

The people of Four Seas have always adhered to their mission and vision. Keeping up with market trends, Four Seas walks steadily and firmly to carry out business reform and upgrade. In the future, the people of Four Seas will break traditional shackles, keep upgrading services, explore with a down-to-earth attitude, and work jointly with partners to realize sustainable brand development.
