# Supplementary material for: Sustainability of translator training in higher education
Source: PLoS One. 2023 May 16;18(5):e0283522. doi: 10.1371/journal.pone.0283522 (PMC10187915; doi:10.1371/journal.pone.0283522)
Supplement: S2 Appendix — (PDF) [file pone.0283522.s003.pdf]

A Questionnaire about Transcreation in the Translation Course

Latest Update

2022-07-01 17:49

Number of questionnaire copies

71

Geographic Locations

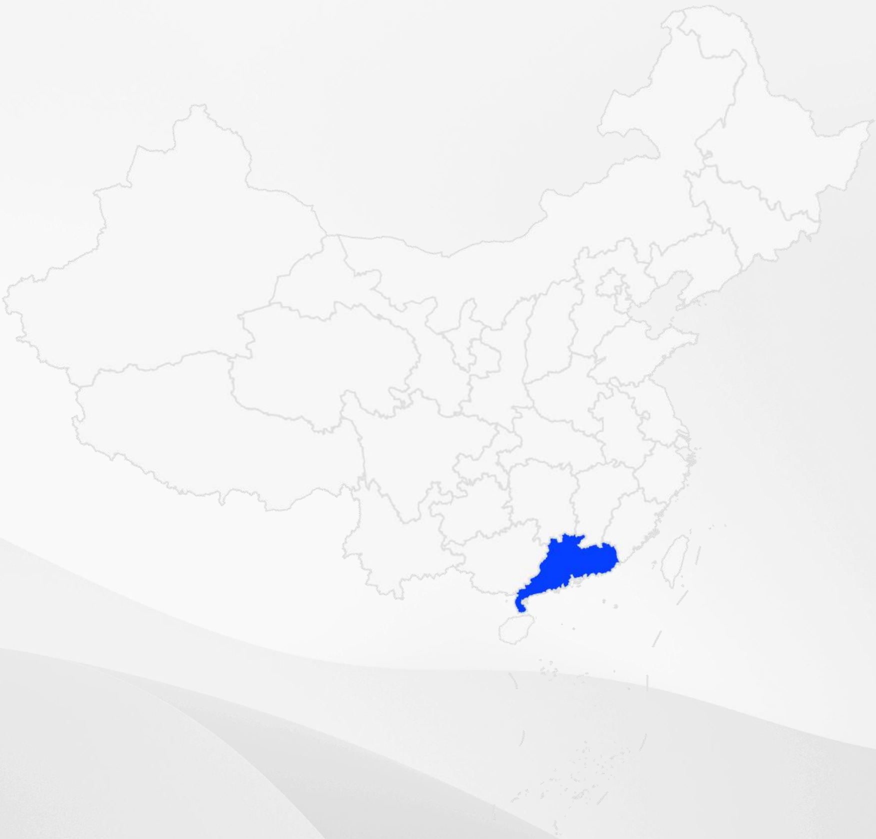

1. No personal identifying information is to be collected in this questionnaire. All the data, if used in future acade mic research, will be anonymous.Doyou agree to give your consent to their use for pure academic purposes?

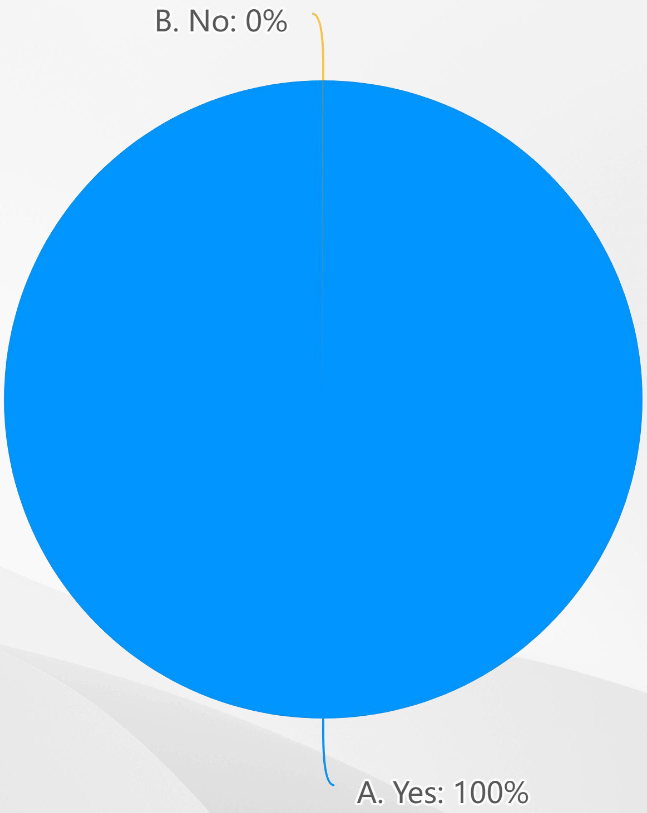

2.Gender

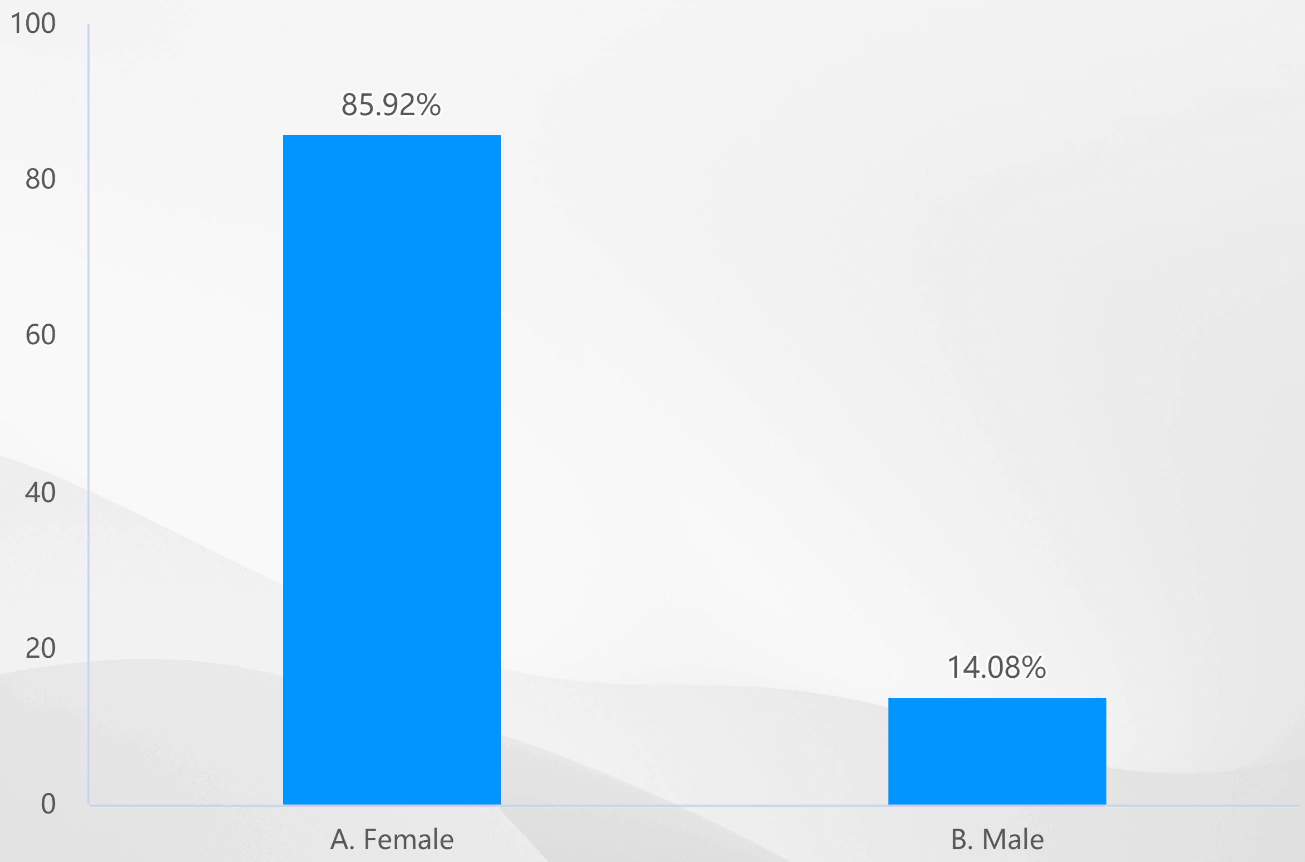

3.Class No.

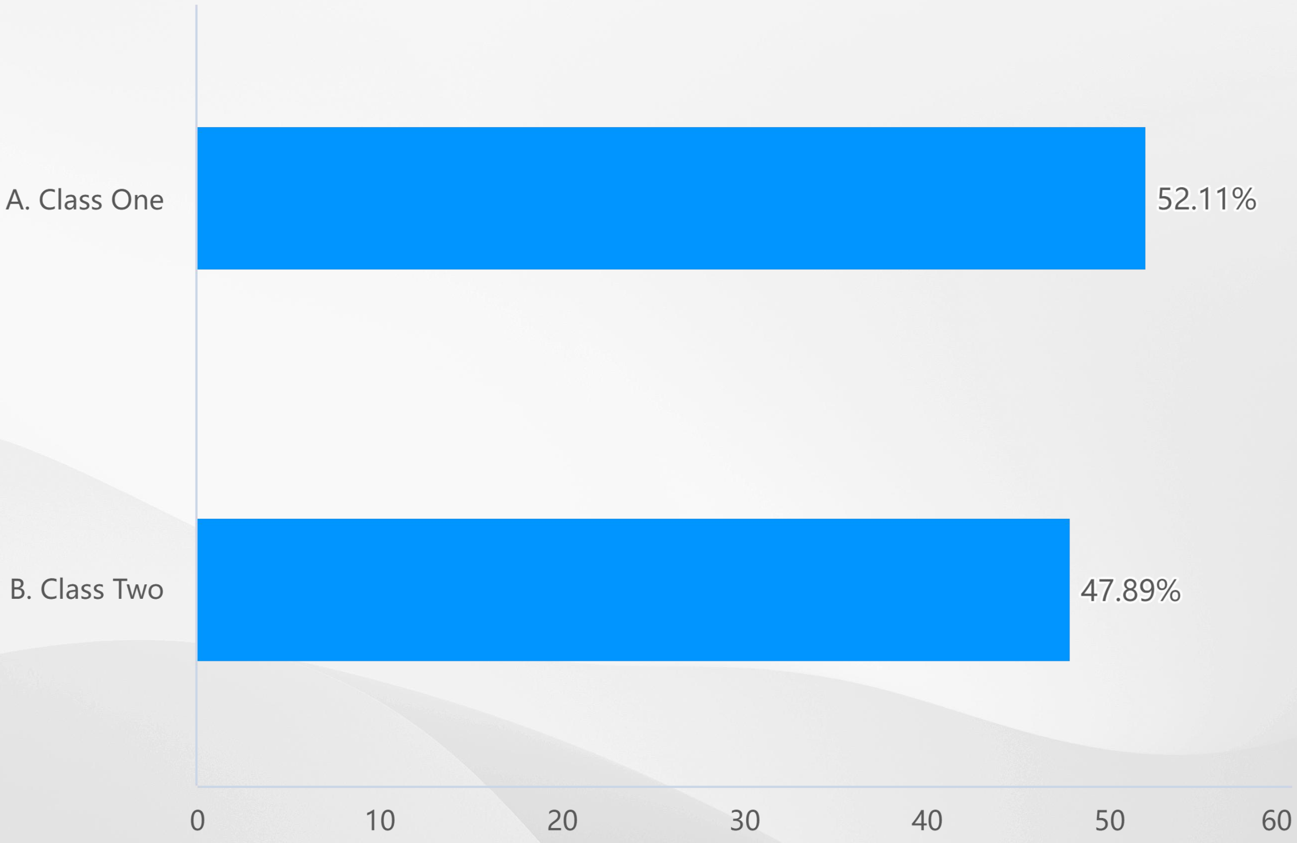

4.This course enables me to have some idea about transcreation.

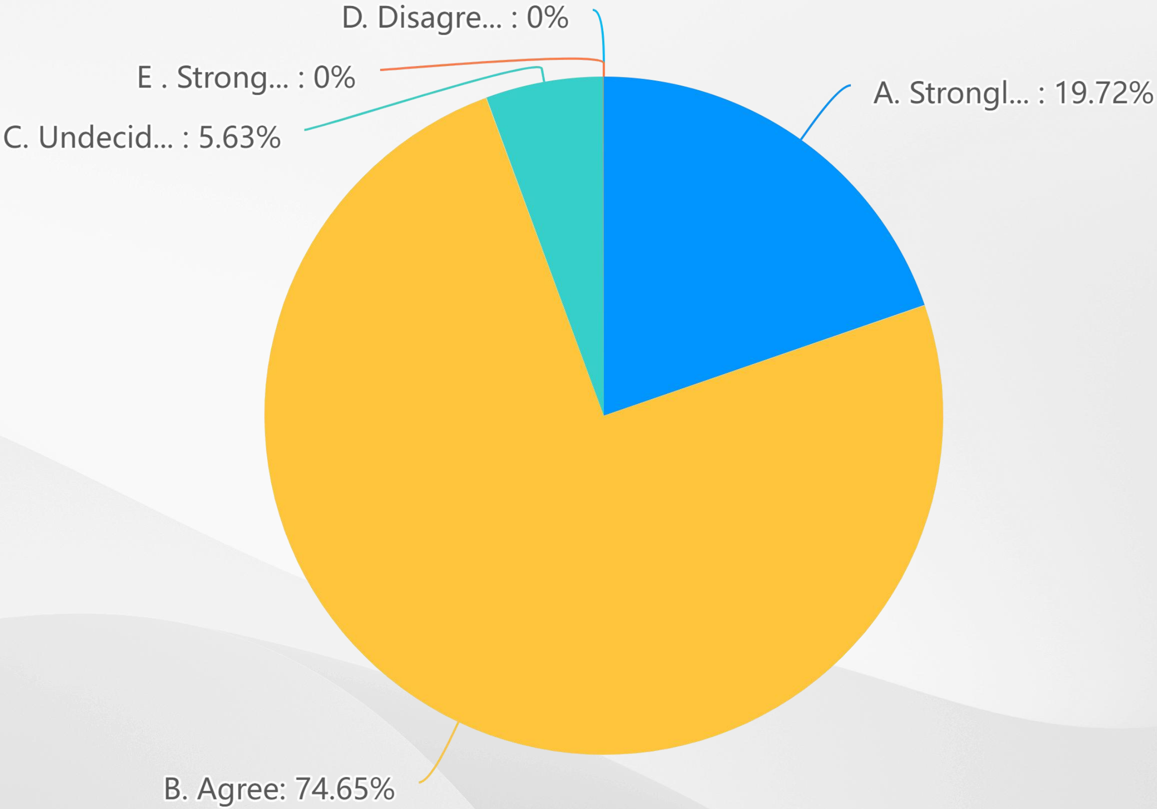

5.Transcreation, a form of rewriting or copy-writing, deviates, to a lesseror greater extent, or even completely, from the source text, so as to better serve the target audience.

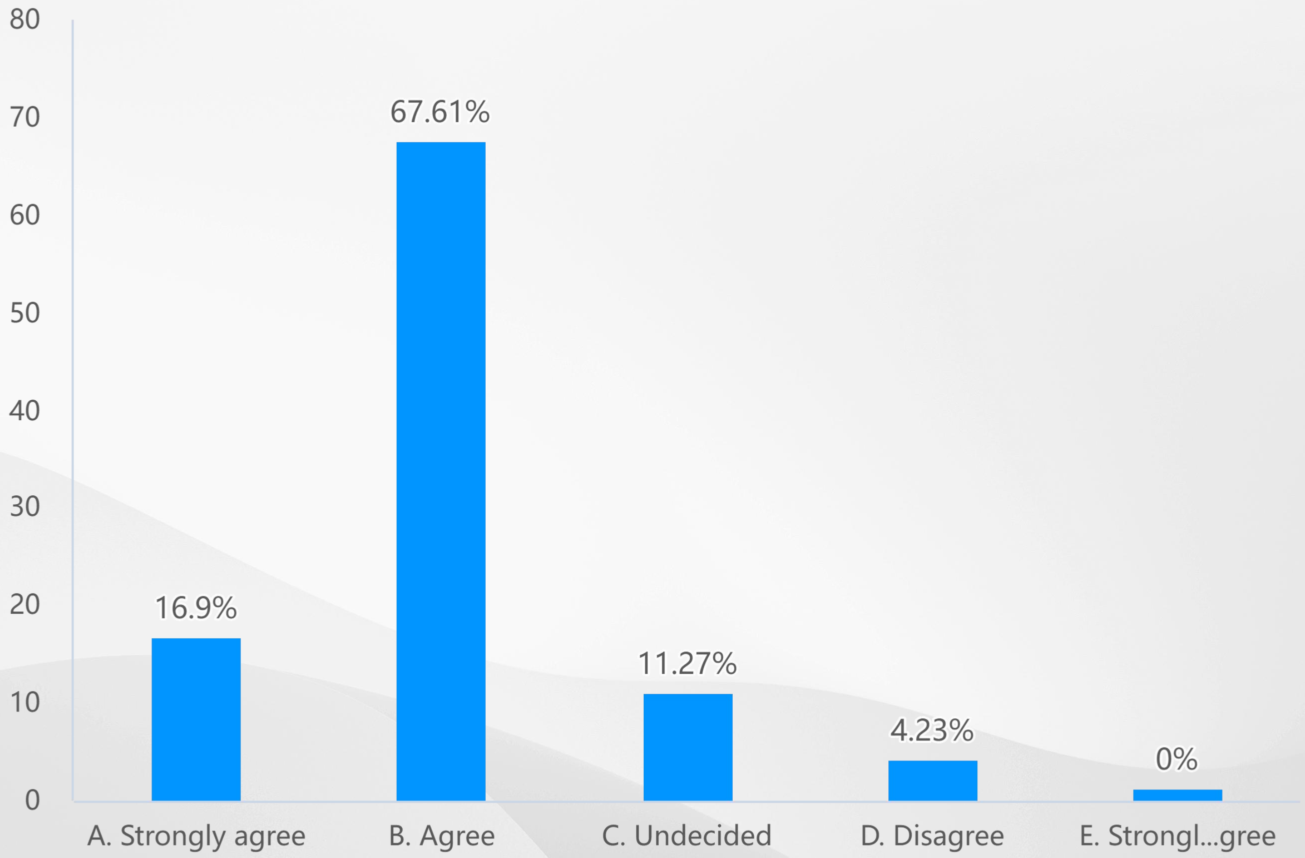

6.This course enables me to understand that human creativity can never be replaced by AI or machine translation when it comes to cross-cultural promotional especially advertising and marketing and other communicative purposes.

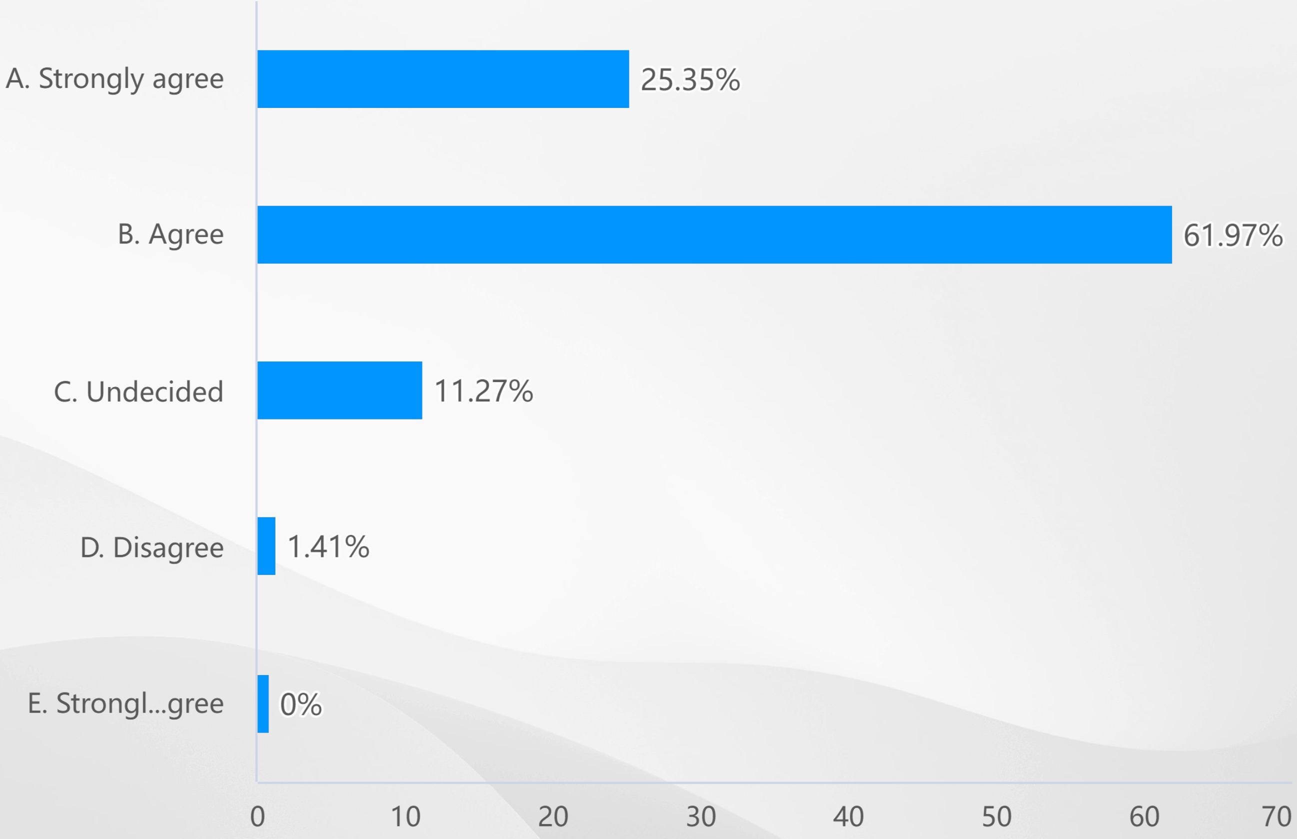

7.Whether to adopt transcreation depends on the skopos or purpose of the translation involved.

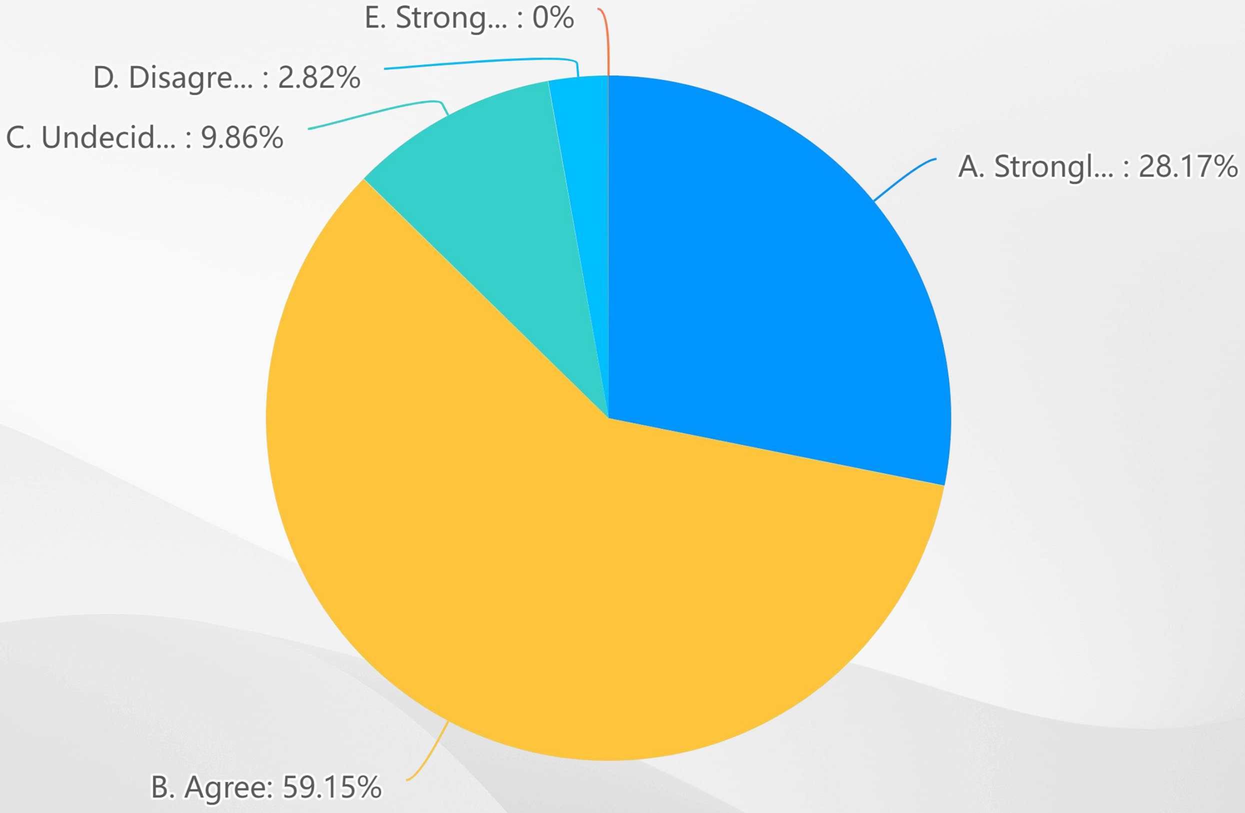

8.In translation, the end (skopos or purpose) justifies the meansincluding but not limited to transcreation .

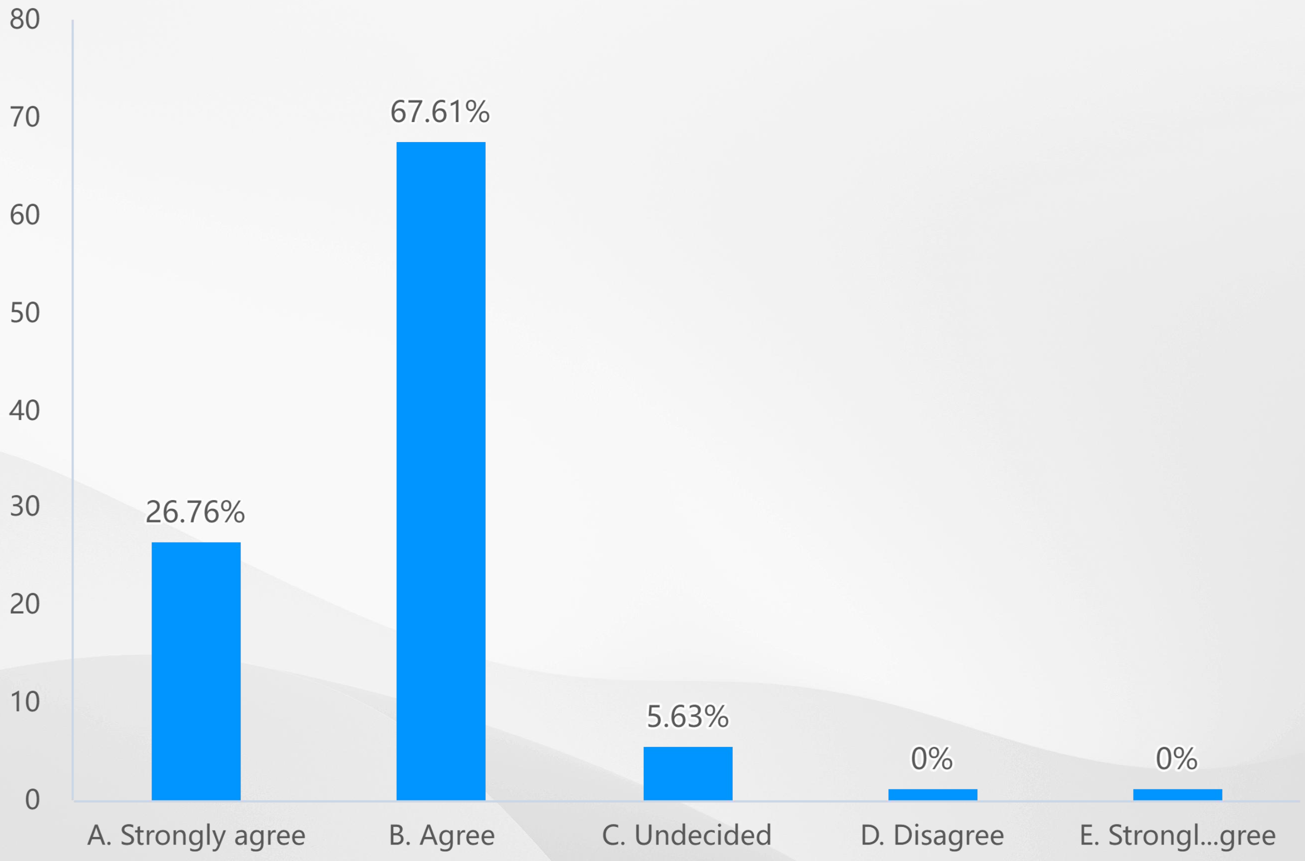

9.In the age of AI, transcreation is a core competence or skill fortranslators when most of conventional translation is taken over by AI or machine translation.

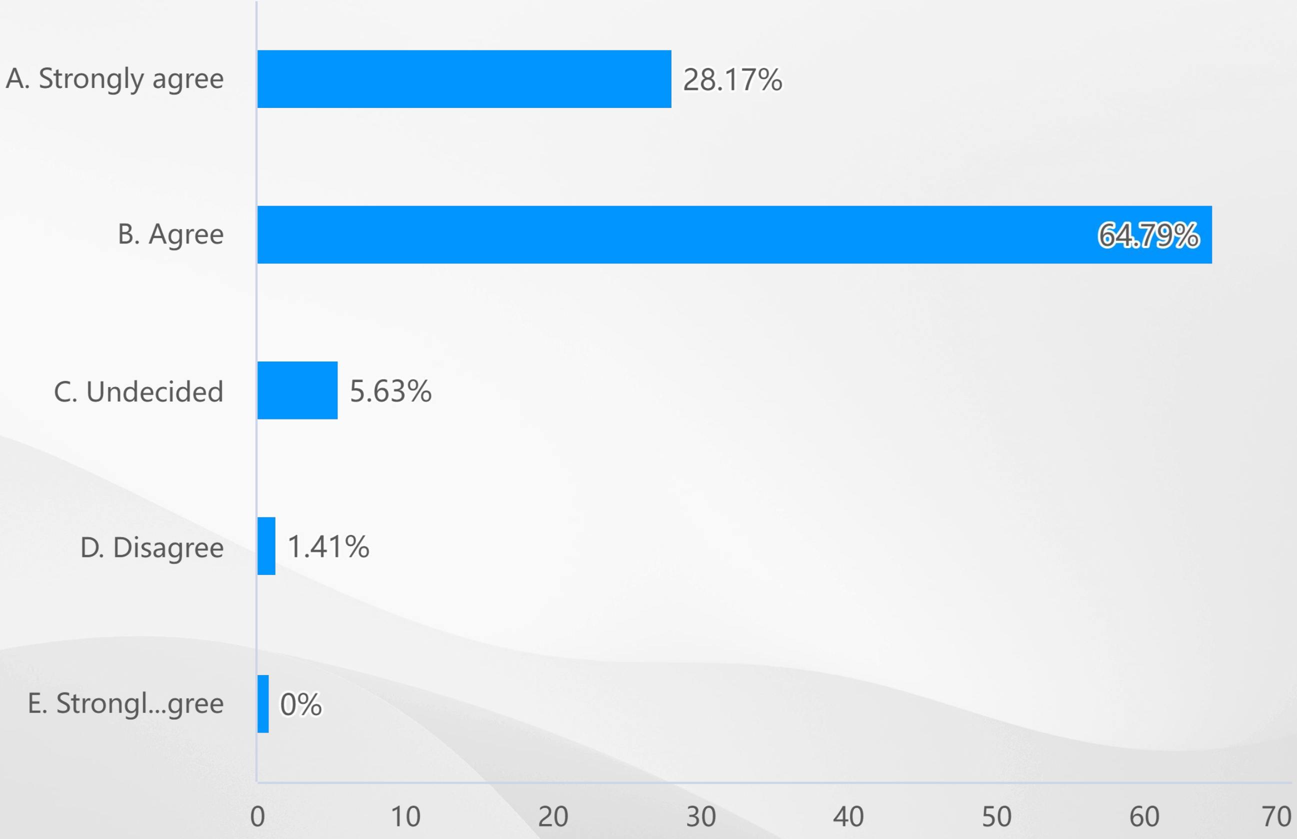

10.The popularity of AI or machine translation means a greatopportunityfor those with skills in transc reation or copy-writing.

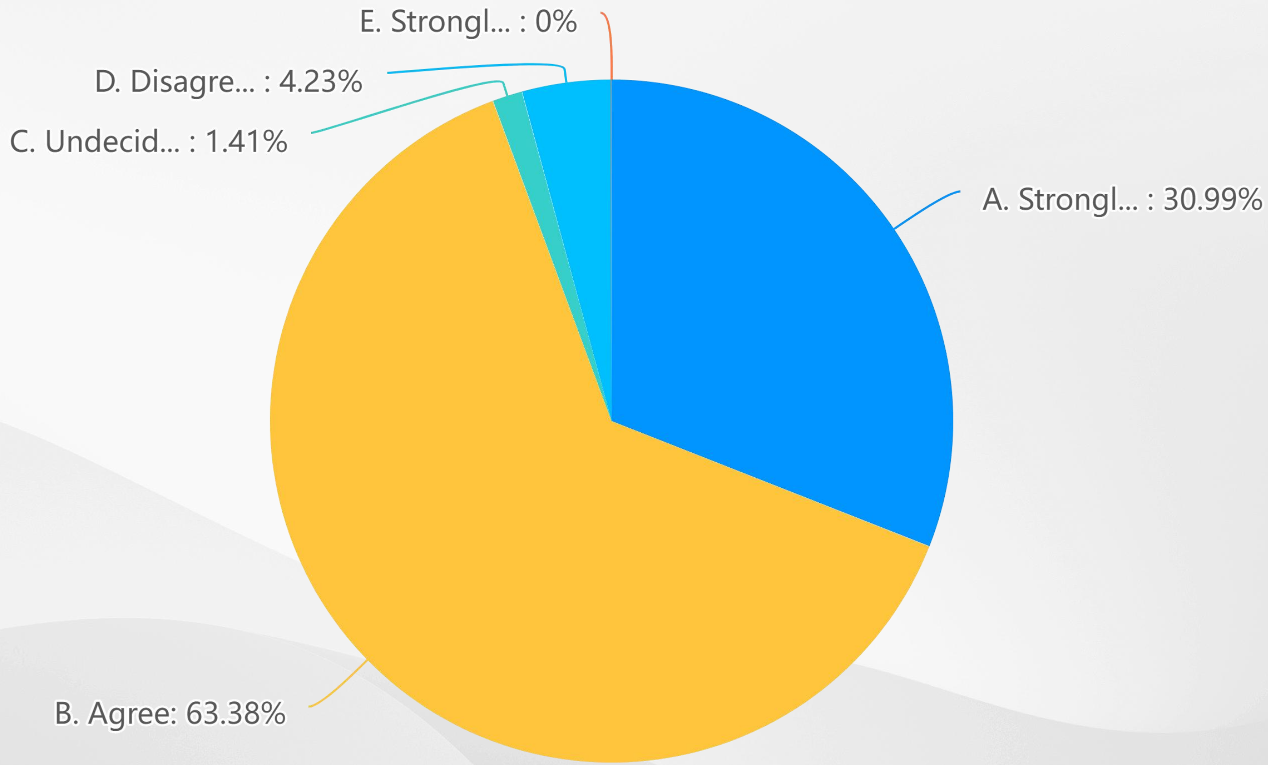

11.This course has boosted my competitiveness or employability tosome degree as a would-be translator.

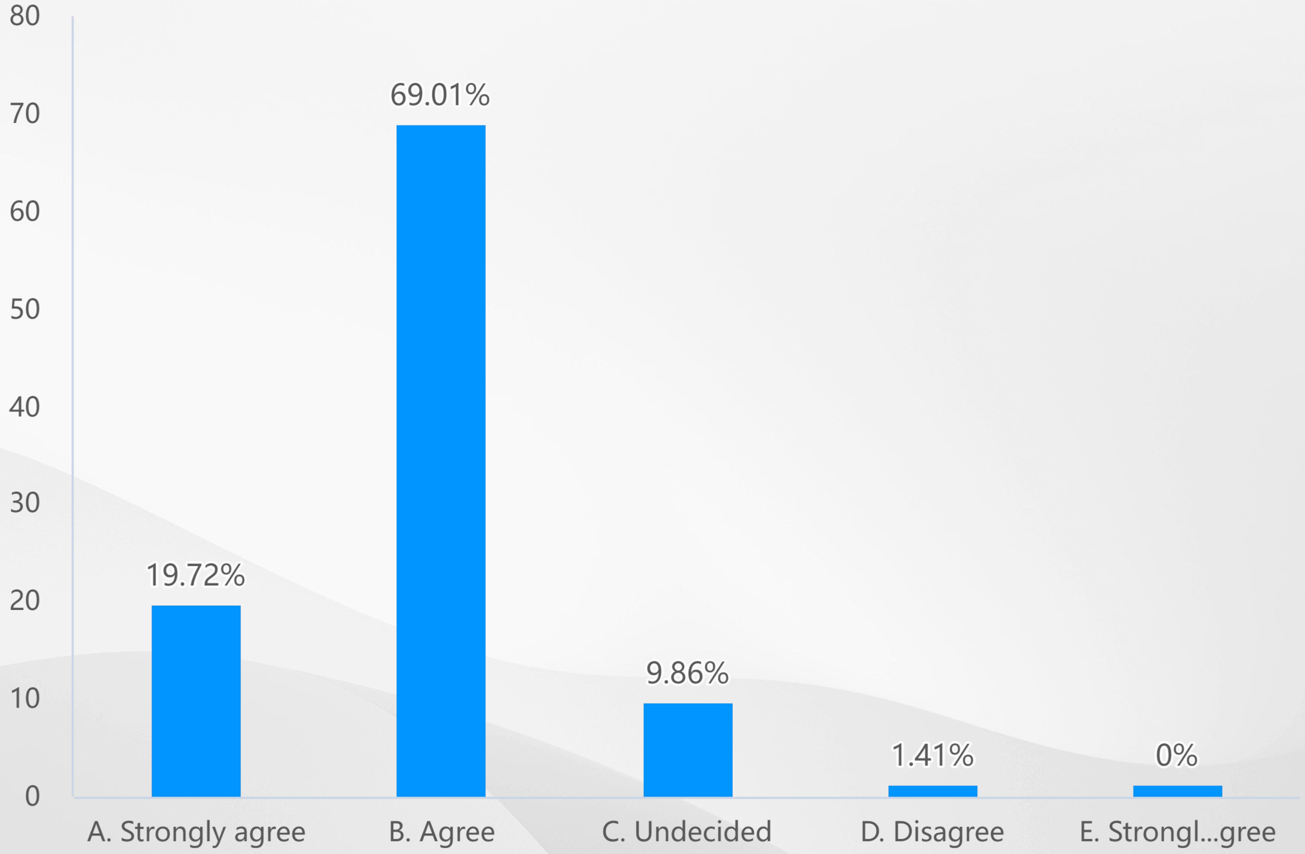

12.The prospects of the job market in the translation industry are brightthough facing huge challenges.

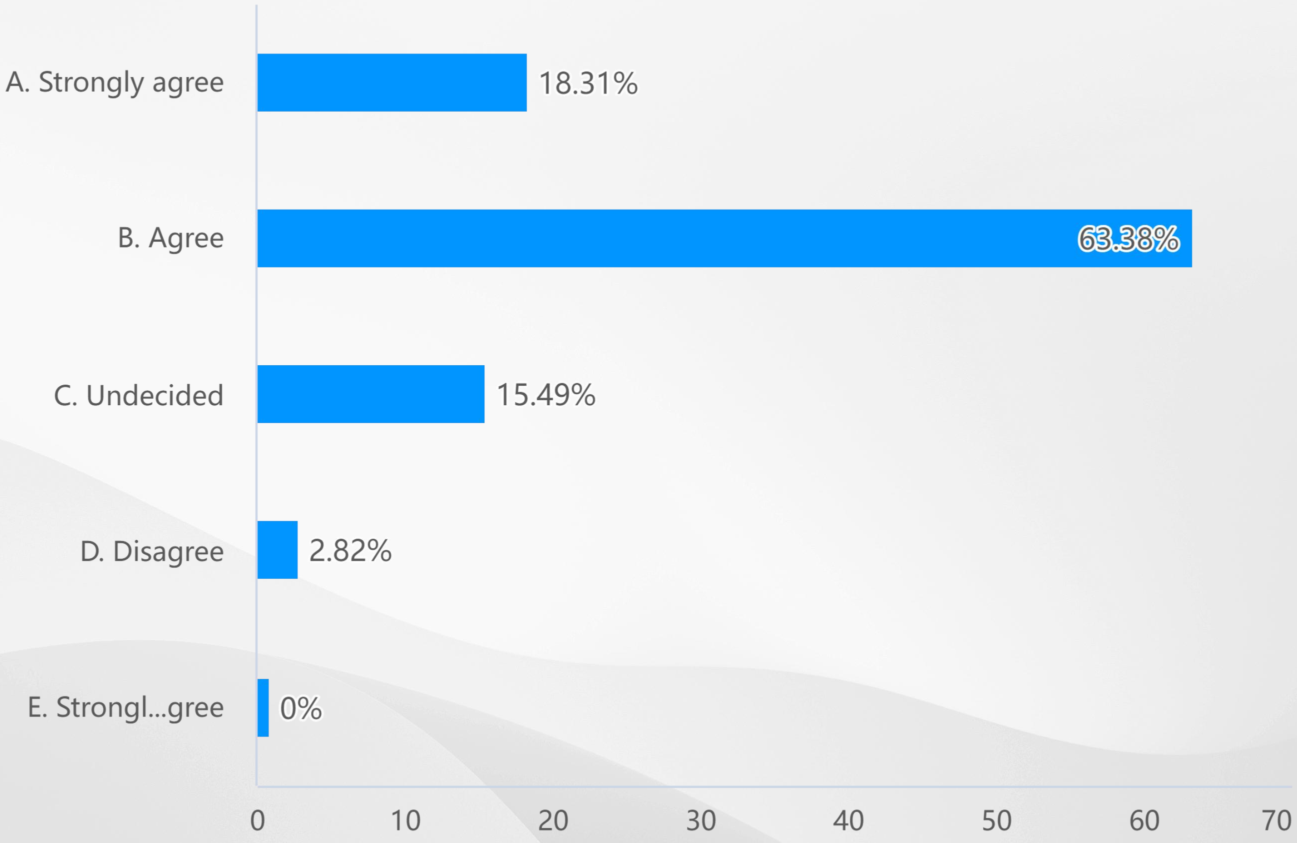

13.The effects of transcreations by students are hard to assess since themarket has the final say even i f translation teachers or clients areimpressed.

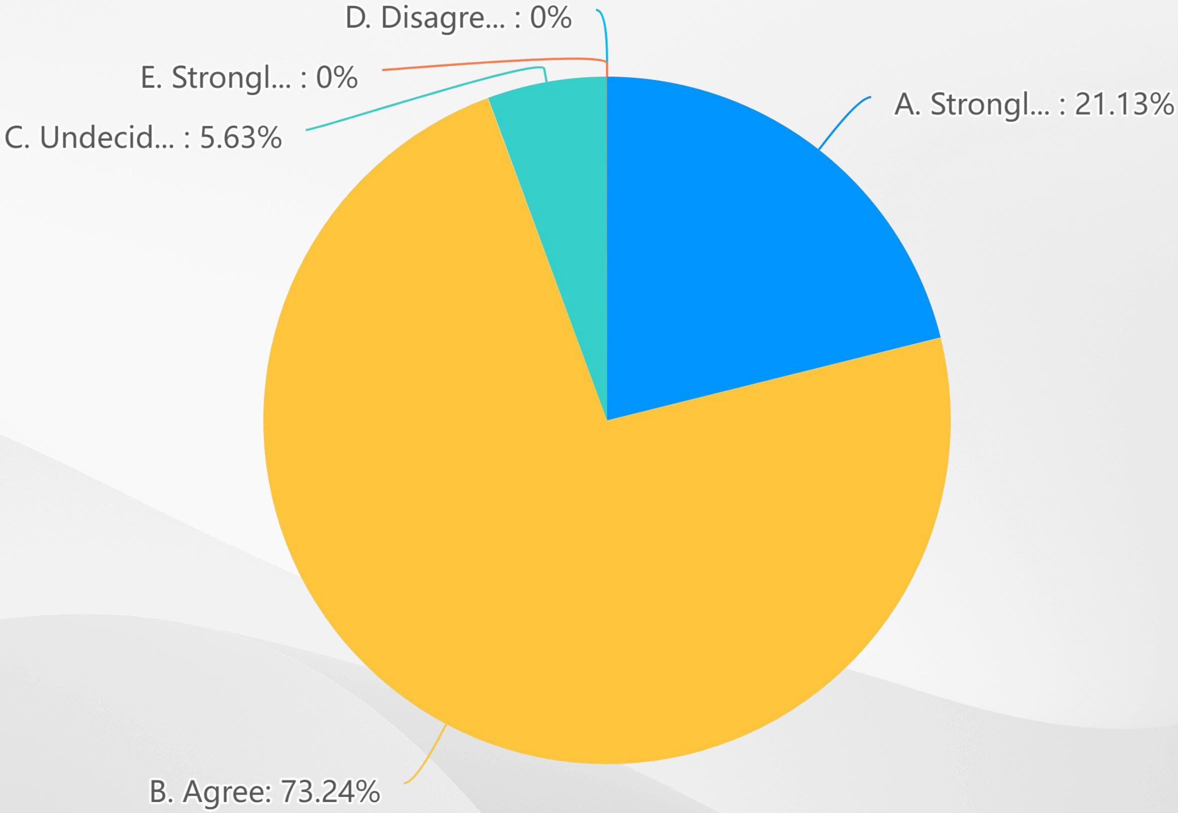

14.Have you any other comments on or suggestions for this course?

I think translation alone is more important than transcreation.More practical translation is desirable.Translation never has the right answer.

The course is OK.

I hope the course can integrate more with the market.

More extra-curricular materials can be incorporated into classroom learning.

Hope we can be exposed to various approaches.

Talk more about the translation in the textbook.
