# Supplementary material for: Sustainability of translator training in higher education
Source: PLoS One. 2023 May 16;18(5):e0283522. doi: 10.1371/journal.pone.0283522 (PMC10187915; doi:10.1371/journal.pone.0283522)
Supplement: S1 File — (PDF) [file pone.0283522.s005.pdf]

Reliability Statistics (Cronbach Alpha)

Items	Corrected Item-Total Correlation(CITC)	Cronbach Alpha if Item Deleted	Cronbach a
4. This course enables me to have some idea about transcreation.	0.551	0.862	0.871
5. Transcreation, a form of rewriting or copy-writing, deviates, to a lesser or greater extent, or even completely, from the source text, so as to better serve the target audience.	0.404	0.874	
6, This course enables me to understand that human creativity can never be replaced by AI or machine translation when it comes to cross-cultural promotional especially advertising and marketing and other communicative purposes.	0.387	0.875	
7. Whether to adopt transcreation depends on the skopos or purpose of the translation involved.	0.716	0.847	
8. In translation, the end (skopos or purpose) justifies the means including but not limited to transcreation.	0.617	0.857	
9、In the age of AI, transcreation is a core competence or skill for translators when most of conventional translation is taken over by AI or machine translation	0.794	0.842	
10. The popularity of AI or machine translation means a great opportunity for those with skills in transcreation or copy-writing.	0.595	0.858	
11. This course has boosted my competitiveness or employability to some degree as a would-be translator.	0.659	0.853	
12.The prospects of the job market in the translation industry are bright though facing huge challenges.	0.612	0.857	
13. The effects of transcreations by students are hard to assess since the market has the final say even if translation teachers or clients are impressed.	0.637	0.856	

Cronbach a (Standardized): 0.875

Suggestions

Reliability analysis is used to study the reliability and accuracy of quantitative data (especially attitude scale questions);

Firstly,Alpha coefficient is analyzed. If this value is higher than 0.8, the reliability is high. If this value is between 0.7 and 0.8, the reliability is good; If this value is between 0.6 and 0.7, the reliability is acceptable; If this value is less than 0.6, that indicates poor reliability;

Secondly,if the CITC value is lower than 0.3, consider deleting this item;

Thirdly, if "Alpha coefficient after deleting an item" is obviously higher than the current Alpha coefficient , we can consider deleting the item and reanalyzing it.

Fourthly,Summarize the analysis.

Intelligent Analysis

It can be seen from the above table that the reliability coefficient value is 0.871, which is greater than 0.8, thus indicating that the reliability of the research data is high.For the "Alphacoefficient afterdeleting an item", the reliability coefficient will not increase significantly after any item is deleted.Therefore, it shows that the item should not be deleted.

As for Item 6 ▫ This course enables me to understand that human creativity can never be replaced by AI or machine translation when it comes to cross-cultural promotional especially advertising and marketing and other communicative purposes, its CITC value is less than 0.4. When making predictions ï

You can correct this item before collecting the data (in formal data analysis, you can either delete this item or keep it). To sum up, the reliability coefficient value of the research data is higher than 0.8, which comprehensively shows the reliability of the data is high and can be used for further analysis.

Cronbach Alpha

N of Items	*n*	Cronbach a
10	71	0.871

References

[1] The SPSSAU project (2022). SPSSAU. (Version 22.0) [Online Application Software]. Retrieved from https://www.spssau.com.

[2] Eisingar R, Te Grotenhuis M,Peizer B.The reliability ofa two- item scale: Pearson, Cronbach or Spearman Brown? [J] International Journal of Public Health, 2013,58 (4): 637-642.

[3] Zhou Jun. Questionnaire data analysis:Exploring six analysis approaches of SPSS [M]. Electronic Industry Press, 2017.
