# Supplementary material for: Sustainability of translator training in higher education
Source: PLoS One. 2023 May 16;18(5):e0283522. doi: 10.1371/journal.pone.0283522 (PMC10187915; doi:10.1371/journal.pone.0283522)
Supplement: S2 File — (PDF) [file pone.0283522.s006.pdf]

Validity Analysis Sorting

Items	Factor Loadings		Communalities
	Factor 1	Factor 2	
4、 This course enables me to have some idea about transcreation.	0.253	0.825	0.745
5、Transcreation, a form of rewriting or copy-writing, deviates, to a lesser or greater extent, or even completely, from the source text, so as to better serve the target audience.	0.124	0.758	0.590
6、This course enables me to understand that human creativity can never be replaced by AI or machine translation when it comes to cross-cultural promotional especially advertising and marketing and other communicative purposes.	0.160	0.659	0.460
7、Whether to adopt transcreation depends on the skopos or purpose of the translation involved.	0.857	0.118	0.749
8、In translation, the end (skopos or purpose) justifies the means including but not limited to transcreation.	0.683	0.215	0.512
9、In the age of AI, transcreation is a core competence or skill for translators when most of conventional translation is taken over by AI or machine translation	0.798	0.366	0.770
10、The popularity of AI or machine translation means a great opportunity forthose with skills in transcreation or copy-writing.	0.785	0.019	0.617
11、This course has boosted my competitiveness or employability to some degree as a would-be translator.	0.685	0.316	0.569
12、The prospects of the job market in the translation industry are bright though facing huge challenges.	0.720	0.145	0.540
13、The effects of transcreations by students are hard to assess since the market has the final say even if translation teachers or clients are impressed.	0.662	0.346	0.557
Eigenvalues (Initial)	4.830	1.278	-
% of Variance (Initial)	48.297%	12.782%	-
% of Cum. Variance (Initial)	48.297%	61.080%	-
Eigenvalues (Rotated)	3.984	2.124	-
% of Variance (Rotated)	39.839%	21.241%	-
% of Cum. Variance (Rotated)	39.839%	61.080%	-
KMO	0.827		-
Bartlett's Test of Sphericity (Chi-Square)	336.525		-
df	45		-
p value	0.000		-

Note: Blue indicates that the absolute value of loading is greater than 0.4, and red indicates that the communality is less than 0.4.

Suggestions

Validity analysis is used to study the design rationality of quantitative data (especially attitude scale questions).

Firstly, the KMO value is analyzed; If this value is higher than 0.8, it shows that the research data is very suitable for extracting information (the validity is very good from this perspective); If this value is between 0.7 and 0.8, it means that the research data is suitable for extracting information (reflecting good validity)。If thisvalue is between 0.6 and 0.7, it means that the research data is suitable for extracting information (the validity is fairly good)。if this value is less than 0.6,it meansthat the data is not suitable for extracting information (the validity is not good) (If there are only two items, KMO is 0.5 anyway);

Secondly, analyze the corresponding relationship between items and factors.If the correspondence is basically consistent with the psychological expectation of theresearch, it means that the validity is good;

Thirdly,if the validity is not good, or the corresponding relationship between factors and items is seriously inconsistent with expectations, or the communality value ofan analysis item is lower than 0.4 (sometimes 0.5), you can consider deleting the item。

Fourthly,the common standards for deleting items are: A. the commonality value is lower than 0.4 (sometimes 0.5); B. there is a serious deviation in thecorresponding relationship between analysis items and factor s;

Fifthly, repeat the above four steps from 1 to 4 until KMO reaches the standard; and the corresponding relationship between items and factors is basically consistentwith expectations, which will showgood validity;

Sixthly, summarize the analysis.

Intelligent Analysis

Validity research is used to analyze whether the research item is reasonable and meaningful. Factor analysis is used for validity analysis, and KMO value, commonality, variance interpretation rate value and factor load coefficient value are used respectively.

And other indicators are comprehensively analyzed to verify the validity level of the data. KMO value is used to judge the suitability of information extraction, commonality value is used to exclude unreasonable research items, and variance interpretation rate valueis used to explain information extraction.

The factor load coefficient is used to measure the corresponding relationship between factors (dimensions) and items. From the above table, it can be seen that the commonality values corresponding to all research items are higher than 0.4,indicating that the research item information can be effectively extracted. In addition, KMO value is 0.827, which is greater than 0.6, and the data can be effectively extracted. In addition, the variance interpretation rates of the two factors are 39.839% and 21.241% respectively, and the cumulative variance interpretation rate after rotation is 61.080%>50%.That means the information of interesting research items can be effectively extracted. Finally, please combine the factor load coefficient to confirm whether the corresponding relationship between the factor (dimension) and the research item is consistent with the expectation. If it is consistent, it means it is valid, otherwise it needs to be adjusted again.When the absolute value of factor load coefficient is greater than 0.4, it means that there is a corresponding relationship between options and factors.

[Hint]1.If a research item and a measure(factor)is totally inconsistent with the expectation,we can consider deleting the research item2.If there is no correspondingrelationship between a research item and a measure(factor),you can consid er deleting the research item.

KMO and Bartlett's Test

KMO		0.827
Bartlett's Test of Sphericity	Chi-Square	336.525
	df	45
	P	0.000

Suggestions

When only KMO and Bartlett tests are used for validity, that means the corresponding relationship between dimensions and analysis items, variance explanation rate and so on are ignored.

First, analyze KMO value. If this value is higher than 0.8, it means that it is very suitable for information extraction (from one perspective , it shows very good validity) ¤ If this value is between 0.7 and 0.8, it means t hatit is suitable for information extraction (from one perspective,that shows the validity is good) ¤ If this value is between 0.6 and 0.7, it means that information can be extracted (from one perspective ,the validity is fairly good) ¤ If this value is less than 0.6, it means that the information is difficult to extract (the validity is low) ¤

Secondly, the validity analysis requires that it should pass the Bartlett test (the corresponding *P* value should be less than 0.05);

Thirdly,if there are only two analysis items, KMO is 0.5 anyway.

Intelligent Analysis

The KMO and Bartlett tests are used to verify the validity. From the above table, it can be seen that the KMO value is 0.827 and the KMO value is greater than 0.8, so the research data is very suitable for extracting in formation (the validity is verygood).

References

[1] The SPSSAU project (2022). SPSSAU. (Version 22.0) [Online Application Software]. Retrieved from https://www.spssau.com.

[2] Chung R H, Kim B S, Abreu J M. Asian American multidimensional acculturation scale: development, factor analysis, reliability, and validity.[J].Cultur Divers Ethnic Minor Psychol, 2004,10(1):66-80.

[3] Zhou Jun. Questionnaire data analysis: Exploring six analysis approaches of SPSS [M]. Electronic Industry Press, 2017.
