# Supplementary material for: Sustainability of translator training in higher education
Source: PLoS One. 2023 May 16;18(5):e0283522. doi: 10.1371/journal.pone.0283522 (PMC10187915; doi:10.1371/journal.pone.0283522)
Supplement: Attachment — Submitted filename: Sustainability of Translator Training in Higher Ed.docx [file pone.0283522.s007.docx]

This is good effort on the part of the authors and seems to be timely as globalization and digitalization are taking place rapidly.

However, the Abstract and Introduction sections are not easy to read.

Not flowing structure and content. Authors dive into the subjects w/o adequate definition of terms or gradual building the ground stage

Opening paragraph is too technical for the general readers

***Should start like*** : The UN has developed 17 *Sustainable Development Goal* (SDG) due to the need of … and the new situation…. (before mentioning target 4.4 or such..)

Again, the opening sentence/paragraph of the **Abstract** is way too long and complex. Those of us who are not familiar with translators and transcreation, these themes need to be introduced and clarified.

38. To achieve the sustainable development goal in education set by the United Nations for
39. 40  2030, one of whose targets is to substantially increase the number of youth and adults possessing
40. 41  relevant skills, including technical and vocational skills, for employment, decent jobs and entrepre-
41. 42  neurship, students need to be equipped with the core competences identified for the field they spe-
42. 43  cialize in. For student translators, transcreation is a core competence they are expected to acquire.

My Suggestion:

*The United Nations has set a Sustainable Development Goal in education to be met hopefully by 2030 (SDG). One of the target areas is to increase, in a substantial way, the number of youth and adults possessing relevant trainings and proficiencies, including technical and vocational skills for employment, affordable jobs, and decent entrepreneurships. Enrolled students need to be equipped with core competencies suitable for the fields in which they are specializing. Translation is one of these fields of specialty. Thus, for student translators, “transcreation” is a core competency they are expected to acquire and practice.*

For example, I had to look up the word **transcreation** online to exactly understand what the authors mean by it and what are they talking about!?! A brief definition of terminologies is essential to any scholarly document before engaging in any detailed discussion.

This is what I found from Google:

*Transcreation is* ***a fusion of the words “translation” and “creation.”***

*It describes copywriting content in a source text that needs to be made coherent, relevant, etc. in a new language. Sometimes transcreation is also called* ***“creative translation.”*** *Namely, because the content isn't translated word for word.*

The **Conclusion** has more flow and clear insight than the opening & the introduction. I suggest the authors include some of that clear description in this paper early on so the educated reader can have an idea of the purpose and direction of this manuscript.

481. 481  In conclusion, the limitations of a one-shot case study are obvious with no random
482. 482  sampling nor a control group. The focus is not to generalize the findings to the whole
483. 483  population but rather, to deepen our understanding of the particular case involved and
484. 484  come up with a solution to the challenges posed by AI and machine translation, which
485. 485  greatly threaten the sustainability of not merely university programs in translation but the
486. 486  translation industry as a whole. Therefore, the ultimate purpose of this paper, in addition
487. 487  to shedding some light on future research along this line, is to appeal for a shift of the
488. 488  focus in translator training to transcreation so that the employability of student translators
489. 489  can be boosted on the one hand and sustainable development in translation courses and
490. 490  programs can be maintained in institutions of higher education on the other. Future re-
491. 491  search may explore how best to design a translation syllabus and optimize the configura-
492. 492  tion of different modules involving transcreation. Transcreation without a source text can
493. 493  also be probed.

Also seems to me that the author’s mother language is not American-English, thus the discrepancies in the text and lack of flow and connectivity. So, the manuscript can benefit from revision to be made by a couple of English speaking educators who are also familiar with the subject matter.

Too many comas in the text ,,,,,,,, making sentences bit fragmented.

The term Student Translator perhaps should be hyphenated for better reading and conceptualization of this specific target population: student-translator(s) or trainer-translators

Text will better read if authors start some paragraphs with “According to so & so (year), … Rather plugging that phrase at the very end of the sentence, SEE line 79 & 106

The statement of the problem is not clear! Because there are online Apps or engines that engage in translation from local-national into global or international languages, does not necessarily eliminate the need for the human factors (translators) like any other discipline or market item, available in person in tangible forms and also electronically in online forms.

Maybe this very paper has been translated into English with the help of computer software program or similar engine : )

The paper quickly moves to become a **Case Study** - not a discussion/analysis of a major theme topic. Then moves to talk about EDITING on top of the primary subject Translating.

The research method and survey, followed by statistics, then interpretations, all seem reasonable sequences and important steps.

The paper warns against literal translation word for word, verbatim, which is extremely important and commendable.

Then the authors bring up the cross-cultural issues and dynamics, which are extremely important and organic part of any interaction, relationship, and communication: verbal or non-verbal, overt or covert, explicit or implicit.

Here the paper will benefit from citing some major definitions of CULTURE, citing some insights/works in the fields of:

cross-cultural human services,

intercultural & intracultural communication,

layers of multiculturalism,

psychology/sociology of globalization,

links of language-brain-culture,

mentality and tradition,

norms and customs,

social meaning-making,

religion and worldview,

Cultural sensitivity vs counter-culture approaches,

and the like….

Also some literature review needed re: human-machine interaction, experts skills & productivity versus smart machines & computers abilities/productivity. There are a lot of research and insights there.

In the discussion section, it is not clear why the authors focused only on some universities in UK to check whether their MA program incorporates Transcreation, to find only 4 of them do. Since the DGT is the credentialing agency for the EMT in Europe, why not compare that in the whole continent,

It is also common sense that we as trained human students or accomplished experts/scholars alike are aware of the new competition and subtle abilities of computers-soft wares and apps – so we adjust, learn to maneuver, and work in parallel rather one eliminates the other. SO, what is novel about this study and it s finding or recommendation? Not clear!

Some technical notes:

**Text** needs revision for better language, structure, flow, and cleaning of punctuations.

The **tables** and **inserts** within the text are way too many, so I am not sure how professional to keep so many images interrupting the text. Otherwise they need to be reorganized in better way.

The **Reference** section /Bibliography is too short for such a compound subject. Definitely, it is not in APA style yet looks bit crowded.
